# Preferential Release of Newly Synthesized Insulin Assessed by a Multi-Label Reporter System Using Pancreatic β-Cell Line MIN6

**DOI:** 10.1371/journal.pone.0047921

**Published:** 2012-10-25

**Authors:** Ni Hou, Hideo Mogami, Chisato Kubota-Murata, Meng Sun, Toshiyuki Takeuchi, Seiji Torii

**Affiliations:** 1 Institute for Molecular and Cellular Regulation, Gunma University, Maebashi, Gunma, Japan; 2 Department of Genetics and Molecular Biology, Xi'an Jiaotong University School of Medicine, Xi'an, Shaanxi, China; 3 Department of Health and Nutrition Sciences, Hamamatsu University, Hamamatsu, Shizuoka, Japan; 4 National Institute for Physiological Sciences, National Institute of Natural Sciences, Okazaki, Aichi, Japan; Institut Curie, France

## Abstract

Newly synthesized hormones have been suggested to be preferentially secreted by various neuroendocrine cells. This observation indicates that there is a distinct population of secretory granules containing new and old hormones. Recent development of fluorescent timer proteins used in bovine adrenal chromaffin cells revealed that secretory vesicles segregate into distinct age-dependent populations. Here, we verify the preferential release of newly synthesized insulin in the pancreatic β-cell line, MIN6, using a combination of multi-labeling reporter systems with both fluorescent and biochemical procedures. This system allows hormones or granules of any age to be labeled, in contrast to the timer proteins, which require fluorescence shift time. Pulse-chase labeling with different color probes distinguishes insulin secretory granules by age, with younger granules having a predominantly intracellular localization rather than at the cell periphery.

## Introduction

In pancreatic β-cells, insulin is initially synthesized as a precursor protein (prepro-insulin) on the rough endoplasmic reticulum (ER), and is converted to proinsulin upon removal of a signal sequence during co-translational insertion into the ER lumen [1]. After passage through the Golgi complex, proinsulin is transported to the *trans*-Golgi network (TGN) where it is sorted and packed into the immature secretory granules (ISGs). During maturation of ISGs to secretory granules (SGs), bioactive insulin is produced by the catalytic activities of PC1/3, PC2 and carboxypeptidase E [2]. Relatively few SGs undergo exocytosis, with most granules stored for several days, and aged granules are subject to degradation [3]. As such, the population of insulin is not secreted in the order in which it is synthesized as demonstrated by pulse-chase experiments with metabolic labeling of cultured islets, which showed that young insulin is secreted first [4]–[7].

Exocytosis, including SG targeting and membrane fusion, has been the focus of the studies on pancreatic β-cells that responded to glucose stimulation with pulsatile insulin secretion. Capacitance measurements of the cell membrane have been performed to monitor the exocytotic events in single β-cells, and individual SG exocytosis has been imaged directly using recently developed total internal reflection fluorescence (TIRF) microscopy [8]. Recent studies using simultaneous capacitance measurements and TIRF imaging suggested that insulin is stored in distinct SGs based on its age and younger insulin granules (up to 48 hours old) are secreted first from human pancreatic β-cells [9].

Because the methods using fluorescent proteins have the limitation of their own fluorescence time, we used the HaloTag™ interchangeable labeling system [10] to monitor the dynamics of each insulin granule as a function of age. Human insulin fused with HaloTag (HT) was expressed in the mouse β-cell line MIN6, and was covalently labeled with specific probes via the HT-tag. Pulse-chase experiments using multiple probes revealed that newly generated insulin granules at non-peripheral locations are preferentially released in response to glucose stimulation.

**Figure 1:**
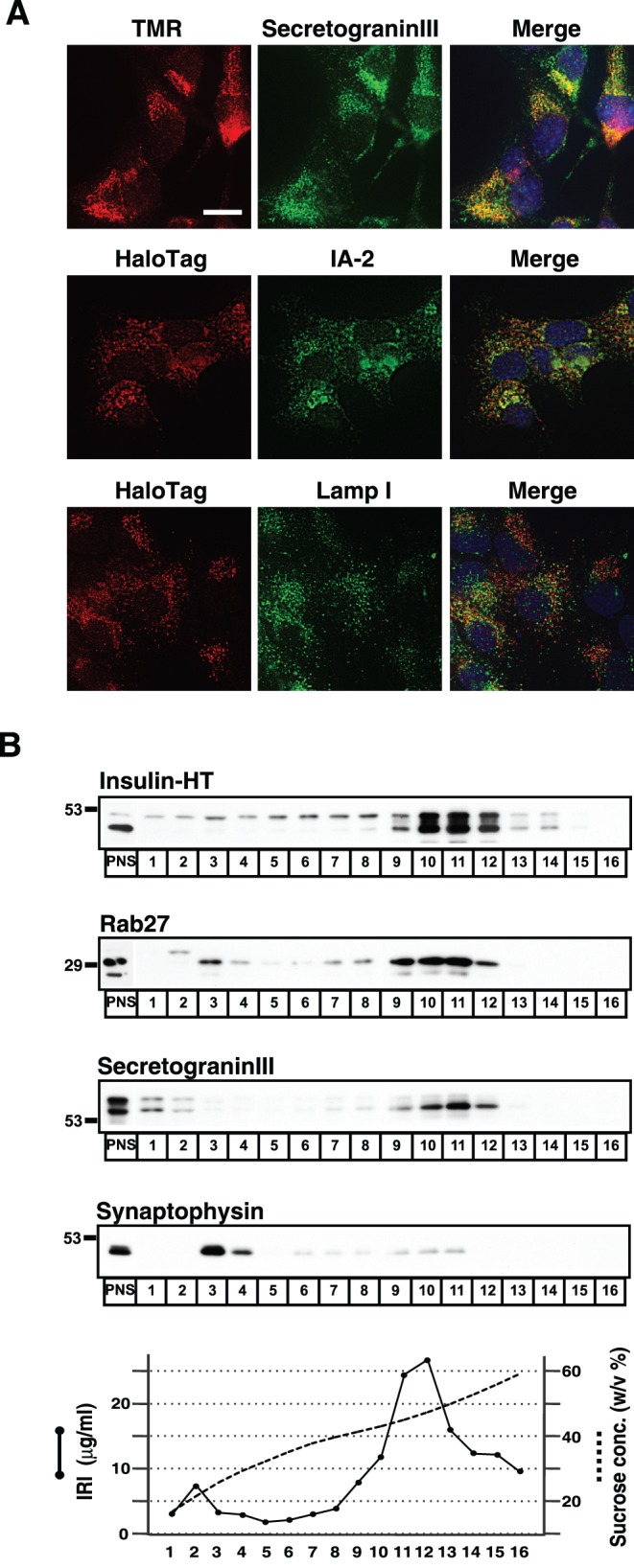
Insulin-HT targets to secretory granules in MIN6 cells. A, MIN6 cells stably expressing insulin-HT (clone #67) were incubated with 5 µM HT-TMR probe for 30 minutes. The cells were then fixed and stained with anti-secretogranin III and Alexa488-conjugated anti-rabbit IgG antibodies (top panels). The stable cells were double-immunostained with anti-HaloTag and anti-IA-2 antibodies (middle panels) or anti-HaloTag and anti-lamp I antibodies (bottom panels). DAPI staining was performed for indentifying nucleus. Fluorescent images were captured by confocal microscopy. Bar, 10 µm. B, MIN6/insulin-HT cells were homogenized and the post-nuclear supernatants (PNS) were separated on a linear sucrose density gradient. Sixteen fractions were collected, and equal volumes from each were analyzed by immunoblotting with antibodies for HaloTag, rab27A/B, secretogranin III, or synaptophysin. Immunoreactive insulin (IRI) in a portion of each fraction was measured.

## Methods

### Ethics Statement

We do not require an ethics statement.

### Plasmid Construction

Full length human insulin were amplified by polymerase chain reaction (PCR) using insulin-enhanced green fluorescent protein (EGFP) [11] as a template and were subcloned into the *Nhe*I and *Xho*I sites of pFC14A (HaloTag7) CMV-Flexi vector (Promega, Madison, WI). The fragment of insulin-HaloTag (insulin-HT) with the *Nhe*I and *Xba*I termini generated by PCR was subcloned into the pcDNA3 vector (Invitrogen, Carlsbad, CA). All subcloned PCR products were confirmed by DNA sequencing. Recombinant adenovirus was constructed as described previously [12].

**Figure 2:**
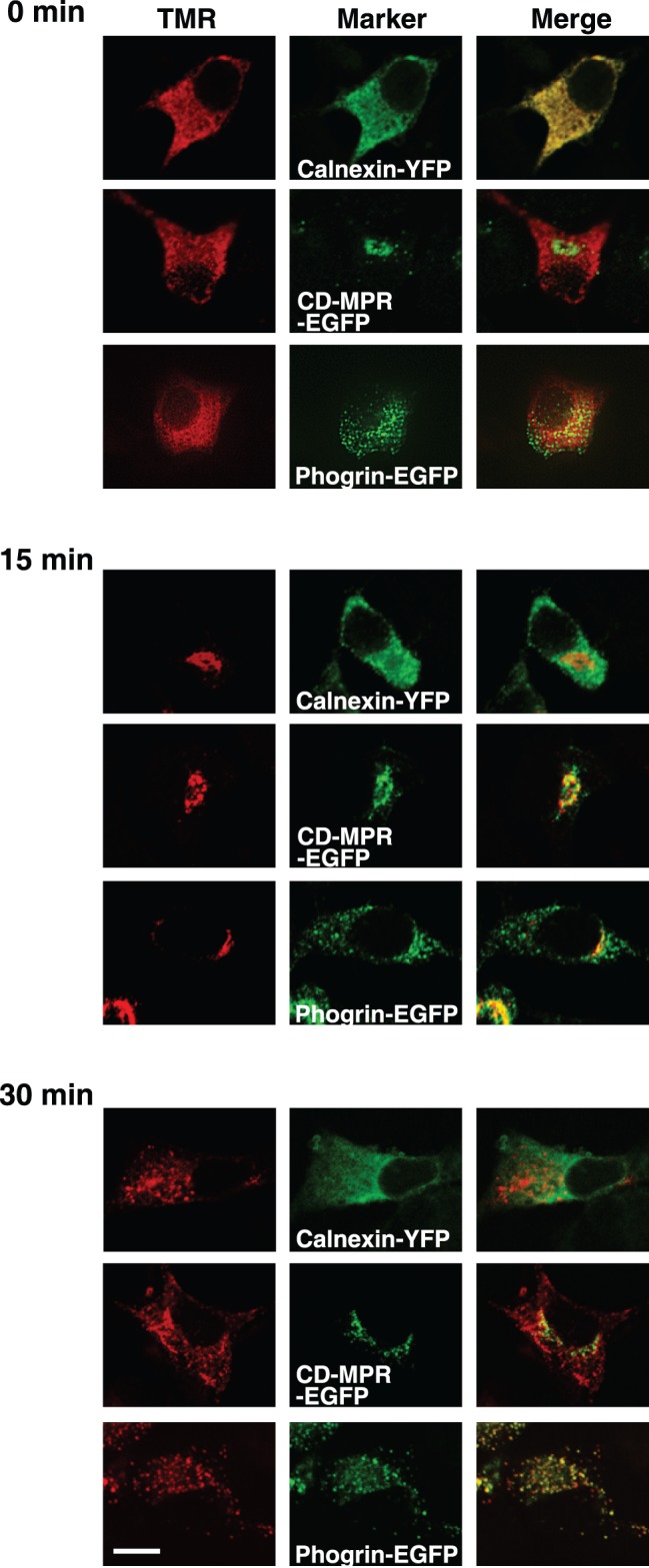
Biosynthetic pathway of insulin-HT monitored by fluorescent microscopy. MIN6 cells transiently transfected with insulin-HT plus calnexin-YFP, insulin-HT plus M6PR-EGFP, or insulin-HT plus phogrin-EGFP were treated with blocking probe for 1 hour. After removal of excess probe, the cells were labeled with HT-TMR for 1 hour at 15°C (top panels). The cells were subsequently chased at 37°C for 15 (middle panels) and 30 minutes (bottom panels), respectively. Fluorescent images were analyzed by confocal microscopy. Experiments were repeated three times with reproducible results. Bar, 10 µm.

### Stable Cell Line Construction

Transfections were performed with Lipofectamine 2000 reagent (Invitrogen). Mouse pancreatic β-cell line MIN6 [12] were transfected with pcDNA3-insulin-HT and stable clones were selected in the presence of G418. Isolated colonies of the insulin-HT-transfected cells (a total of 72 clones) were transferred to new culture dishes for propagation and further selected by fluorescence analysis with HT-tetramethylrhodamine (TMR) probe. Although our radioimmunoassay for insulin does not distinguish the insulin-HT from endogenous insulin, the insulin content of the MIN6/insulin-HT stable cells (clone #67) was approximately 0.37±0.3 nmol/10^6^ cells, which was 46% lower than that found in the parental MIN6 cells (0.69 nmol/10^6^) [13]. The percentage of proinsulin found in stable cells was similar to the percentage of proinsulin found in the culture medium of the MIN6 cells (18% versus 15%). Glucose-responsive insulin secretion was measured as previously reported [12]. When the glucose concentration was raised from 2 mM to 25 mM, the stable cells showed nearly a 2.7-fold increase in insulin secretion (2.6% of content in response to 25 mM glucose for 30 min).

**Figure 3:**
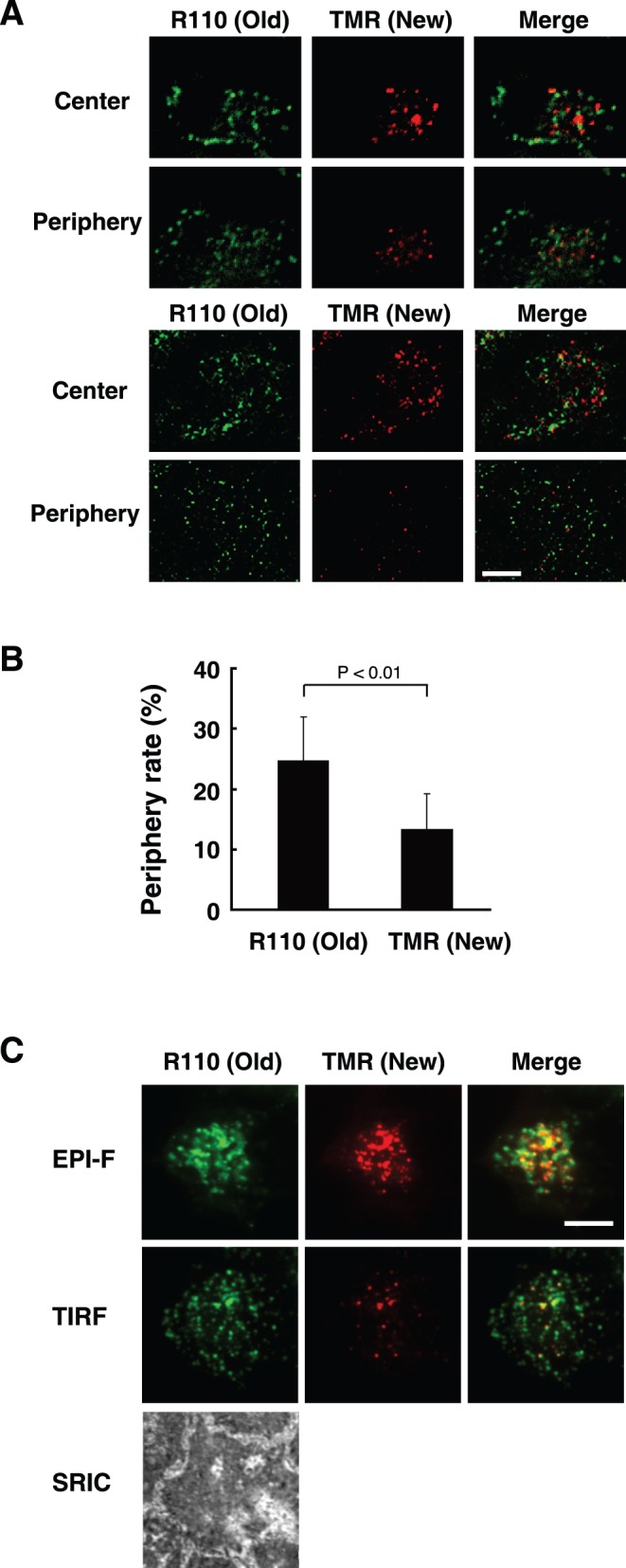
Secretory granules containing newly synthesized insulin are located at the interior of the cell. A, MIN6/insulin-HT cells were incubated with 100 nM HT-R110 probe for 16 hours. The cells were then incubated in modified Krebs-Ringer buffer lacking dyes for 1 hour, and further incubated with 5 µM HT-TMR in buffer for 30 minutes. After removal of excess probe, the cells were fixed and fluorescent signals of R110 and TMR were observed by confocal microscopy in sequential z-axis planes. The periphery and the center images (upper panels) were from stage 2 and 9, respectively (full images were shown in Fig. S3). Another set of cell picture was shown in the lower panels. Bar, 10 µm. B, Intracellular localization of new or old insulin-HT was analyzed as in (A). Their periphery rates were quantified as described in *Methods*. Data are shown as the mean ± SEM of six independent experiments. C, Cells treated as in (A) were analyzed by total internal reflection fluorescence (TIRF) microscopy. Epifluorescence (non-confocal), TIRF, and surface reflection interference contrast (SRIC) images were simultaneously captured. Experiments were repeated three times with reproducible results. Bar, 10 µm.

### Fluorescence Microscopy

MIN6/insulin-HT stable cells were incubated in the dark at 37°C with 5 µM HT-TMR probe for 30 minutes, washed with PBS and culture media, and then incubated in media for 30 minutes to remove unbound probe. Plasmids encoding calnexin-yellow fluorescent protein (YFP) (provided by Dr Hatsuzawa), cation-dependent-mannose-6-phosphate receptor (M6PR)-EGFP [14] and phogrin-EGFP [15] were used to observe ER, TGN/ISG, and SG, respectively. Indirect immunofluorescence staining was performed as described previously [15]. Anti-HaloTag rabbit, anti-rab27A/B rabbit, anti-IA-2 mouse, and anti-lamp-I (CD107a) rat antibodies were purchased from Promega, Immuno-Biological Laboratories (Gunma, Japan), Abnova (Taipei, Taiwan), and BD Bioscience (Lexington, KY), respectively. Live or fixed cells were observed with a stage top incubator/microscope system (INU, Tokai Hit, Fujinomiya, Japan), or an FV-1000 (Olympus, Tokyo, Japan) or a LSM5 PASCAL (Carl Zeiss, Jena, Germany) confocal microscope. TIRF microscopy was carried out using an Nikon inverted microscope (Ti-E, Nikon, Tokyo, Japan) equipped with a TI-SFL Epi-FL Illuminator Unit (white light TIRF), a 130 W HG light source (HG Fiber Illuminator, Nikon), an objective lens (CFI Plan Apo TIRF 60X, NA 1.45, Nikon) and an electron multiplier cooled coupled charge device digital camera (C9100-12, Hamamatsu Photonics, Hamamatsu, Japan). The periphery rate of insulin-HT was quantified as follows. Twenty sequential focal plane images were taken along with Z-axis by the confocal microscopy. The periphery images were derived from the top stage that was almost attached to the glass slide. The center images were from the stage 8–10 µm far from the periphery images, to avoid overlap of SGs. Fluorescent signals that showed only a punctate granular pattern were counted as positive. The total number of SG in the center or the periphery image was counted, and its periphery rate (Periphery/Periphery+Center) was quantified. For each experiment, a total of ten cells were unambiguously assessed.

**Figure 4:**
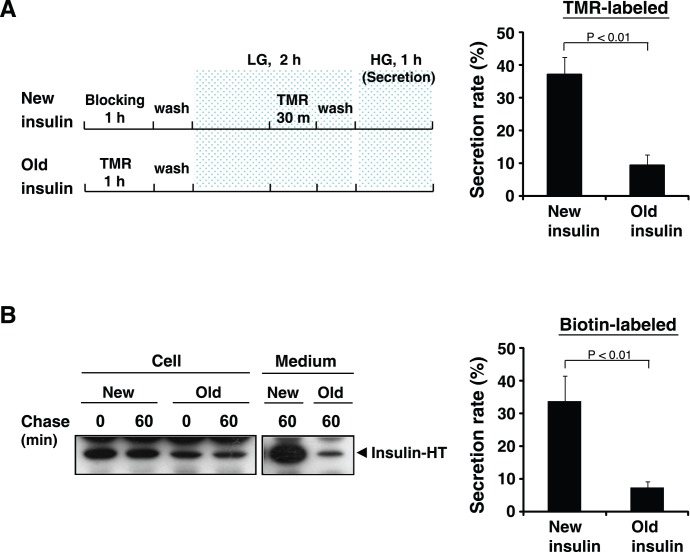
Newly synthesized insulin is preferentially secreted in response to high glucose. A and B, MIN6/insulin-HT cells were labeled with HT-TMR (A) or HT-biotin probe (B) for the indicated time as described in Methods (Scheme is shown in A). The cells were stimulated by high glucose (HG) for 1 hour, and the collected media and cell extracts were analyzed by fluorescence spectrometry (A) or immunoblotting with anti-biotin antibody (B). The intensity of each insulin-HT band was quantified with a densitometric imager. Cumulative data are presented as a percentage of the cellular insulin-HT protein levels at time 0 (before stimulation) as the mean ± SE of three independent experiments.

### Secretion Assay

MIN6/insulin-HT stable cells were at a density of 2×10^6^ cells in 6-cm plate. For labeling newly synthesized insulin-HT, the cells were first treated with blocking probe (10 µM HT-succinimidyl ester (O4) reagent) [16] for 1 hour to mask previously synthesized insulin-HT in the cell. After washing out excess blocking probe, the cells were incubated in modified Krebs-Ringer buffer (2 mM glucose (low glucose: LG), 120 mM NaCl, 5 mM KCl, 24 mM NaHCO_3_, 1 mM MgCl_2_, 2 mM CaCl_2_, 15 mM HEPES (pH 7.4), 0.1% bovine serum albumin) for 1 hour, and further incubated with HT probe (either 5 µM TMR or 1 µM HT-biotin for fluorescence and biochemical analysis, respectively) in buffer for 30 minutes. After washing out the unbound probe (30 minutes), the cells were incubated for 1 hour in buffer containing 25 mM glucose (high glucose: HG) to induce insulin release. For old insulin-HT, stable cells were labeled with HT probe first. The cells were then incubated in buffer (LG) for 2 hours, followed by HG stimulation. Intracellular insulin-HT labeled by TMR was extracted with acid-ethanol buffer (70% ethanol, 1.5% HCl). TMR fluorescence in both the intracellular insulin-HT and the released insulin-HT were measured (555 nm excitation, 575 nm emission) using a fluorescence spectrophotometer (Hitachi F-2500, Ibaraki, Japan). Intracellular insulin-HT labeled by biotin was extracted with lysis buffer (20 mM Tris-HCl (pH 7.5), 150 mM NaCl, 1 mM EDTA, 1 mM EGTA, 0.5% TritonX-100, 1 mM phenylmethylsulfonyl fluoride (PMSF), 10 µg/ml aprotinin, 10 µg/ml leupeptin, and 10 µg/ml pepstatin). Secreted insulin-HT labeled by biotin was immunoprecipitated with anti-insulin guinea pig antibody (Sigma, Saint Louis, MO), and the immunoprecipitates and one fifth of the cell lysates were analyzed by immunoblotting with anti-biotin mouse monoclonal antibody (Jackson ImmunoResearch, West Grove, PA). The immunoreactive band intensity was measured by densitometry, quantified using NIH image 1.62 program, and normalized to an indicated sample in the same membrane.

### Data Analysis

Data are expressed as the mean ± standard errors of the mean (SEM) with the number of individual experiments described in the figure legends. Differences between groups were analyzed using Student’s *t* test. P values less than 0.05 were considered statistically significant.

## Results

We created an insulin-HaloTag construct (insulin-HT) in which human insulin was fused with a 33 kDa protein tag. The resulting expression plasmid was transfected into the mouse pancreatic beta cell line MIN6, and the intracellular localization of the fusion protein was first analyzed by transient incubation with the cell membrane-permeable red fluorescent dye HT-TMR. TMR fluorescence of insulin-HT nearly entirely overlapped with green signals arising from insulin-enhanced green fluorescent protein (EGFP) fusion protein (data not shown), whereas TMR signals were not detectable in untransfected cells, suggesting that the fluorescent signal on vesicles was specific. Because DNA transfection into MIN6 cells is not efficient by conventional transfection methods, we attempted to establish insulin-HT-expressing cell lines (*Methods*). Consequently, we established several stable cell lines and confirmed the steady state distribution of insulin-HT. Immunostaining using anti-secretogranin III antibody revealed that insulin-HT labeled by TMR probe colocalizes with this SG-resident protein (Figs. 1A and S1A). Furthermore, immunofluorescence analysis with anti-HaloTag antibody confirmed that insulin-HT primarily targets to IA-2-positive SGs but not to lysosomes (lamp I-positive) in this stable clone (Fig. 1A). To further examine the localization of insulin-HT, we subjected cellular membranes to ultracentrifugation in a sucrose density gradient under conditions that separate the two types of secretory vesicles in beta cells. Insulin-HT primarily appeared in fractions 10 to 12, which corresponded to a peak for insulin immunoreactivity and the distribution of endogenous rab27A/B and secretogranin III but not to synaptophysin (Fig. 1B). These observations suggest that insulin-HT fusion protein specifically targets and localizes to insulin SGs in the stable cells.

To visualize the trafficking pathway of insulin-HT, pulse-chase experiments were performed with the stable cell line. Pre-existing insulin-HT was first masked by blocking probe and the cells were then labeled with TMR at reduced temperature (15°C), conditions where secretory proteins are known to accumulate in the ER. We observed an ER localization of TMR-labeled insulin that was newly synthesized within 1 hour (Fig. S2 and Fig. 2, upper panels). After the temperature was shifted to 37°C, TMR-labeled insulin appeared to transfer from the ER to the Golgi (Fig. S2). At 15 minutes’ incubation at 37°C, insulin-HT was found at the TGN or ISGs where the EGFP-fused M6PR colocalized (Fig. 2, middle panels), and by 30 minutes, most TMR signals showed an identical localization with phogrin on SGs (Fig. 2, lower panels).

We next used two different fluorescent probes, TMR and R110, to distinguish newly synthesized insulin-HT. R110 direct probe is a cell membrane-permeable green fluorescent dye that can label target proteins upon overnight incubation. Following R110 labeling of pre-existing insulin-HT, newly (∼ 1.5 hours) synthesized insulin-HT was labeled by TMR in the stable cells, whereupon fluorescent signals were observed by confocal microscopy. R110-labeled old insulin-HT signals showed a punctate pattern throughout the cytoplasm that largely did not overlap with new insulin-HT TMR signals (Fig. 3A). This result is consistent with previous observations using atrial natriuretic factor-tagged fluorescent timer protein, in which secretory vesicles segregated into distinct populations according to age in bovine adrenal chromaffin cells [17]. In contrast to chromaffin cells, in which young rather than older secretory vesicles preferentially docked to the plasma membrane, newly synthesized insulin-HT TMR signals were rarely observed in sections either from the top and bottom z-axis stages in MIN6 cells, whereas R110 signals from older insulin were present throughout the stages (Figs. 3A and S3, and Fig. S1B). A rough estimate of peripheral localization of TMR or R110 vesicles revealed that the proportion of new SGs was lower than that of old SGs (Fig. 3B). We then employed total internal reflection fluorescence (TIRF) microscopy to confirm this observation. In contrast to R110 signals, TMR-positive signals were very sparse in the evanescent field images obtained by TIRF, but were evident in the epifluorescent images (Fig. 3C). These results suggest that young SGs containing newly synthesized insulin are located inside the cell with few found at the plasma membrane.

We next examined glucose-induced insulin secretion using stable cells and compared newly synthesized insulin-HT with older insulin as a function of the secretion rate. As the R110 probe is insufficient to label all pre-existing insulin-HT, we designed and performed two sets of pulse-chase experiments, using a blocking probe instead of R110 (Methods) (Fig. 4A, left scheme). Pulse-chase TMR-labeled insulin from the culture media and cell lysates was measured by fluorescence spectrophotometry, and the secretion rate in response to high glucose (25 mM) stimulation was compared for newly synthesized insulin-HT and older insulin. By 60 min, 36.9% of newer insulin-HT was released, whereas only 9.3% of the older protein was found in the secretion media (Fig. 4A, right graph). Similar pulse-chase experiments using HT-biotin probe were examined, and biotin-labeled insulin was detected by immunoblotting with anti-biotin antibody (Fig. 4B, left panels, Fig. S4). In this assay, 33.7% of newly synthesized insulin-HT was secreted into the extracellular spaces, with only 7.4% of older proteins being released (Fig. 4B, right graph). Taken together, these data suggest that newly generated insulin SGs in MIN6 cells undergo exocytosis in preference to older SGs.

## Discussion

In the present study, we describe a new method to study intracellular trafficking and stimulus-coupled secretion of peptide hormones, using insulin as a model. Traditional pulse-chase experiments using radioisotopes enabled us to distinguish synthesized hormones according to their age, but did not allow direct visualization of protein age, so whether newly synthesized and older hormones packed into the same or distinct SGs was unclear. Recent studies with bovine adrenal chromaffin cells using a fluorescent timer protein DsRed-E5, which progressively shifts its fluorescence emission from green to red demonstrated that secretory vesicles segregate into distinct populations based on age [17], [18]. In this case, the age of protein cargo or vesicle is completely dependent on the nature of the fluorescent protein. Thus, we chose the multi-labeling reporter system HaloTag™, and coupled pulse-chase experiments with fluorescence imaging. Our system thus allows measurement of hormone secretion events by both classic biochemical assays and imaging analysis. Furthermore, we produced two simultaneous data sets that support the finding that insulin-HT is packaged into distinct SG populations based on age (Fig. 3), and that newly synthesized insulin-HT is preferentially secreted by high-glucose stimulation (Fig. 4).

In pancreatic β-cells, newly generated SGs from the TGN are transported to the fusion sites at the plasma membrane, and not only fusion step of exocytosis but also the mobilization to the cell periphery is induced by high glucose (HG) stimulation [8]. We observed that populations of young SGs are relatively small at the periphery of MIN6 β-cells under resting conditions (Fig. 3). Furthermore, our pulse-chase experiments revealed the preferential release of newly synthesized insulin (Fig. 4), suggesting that young SGs move from the cell interior to fuse with the plasma membrane for glucose-stimulated insulin secretion. In rat insulinoma INS-1E cells and primary mouse islet cells, young SGs are found predominantly at the cell interior rather than at the cell periphery (Figs. S5 and S6), however, we have not yet detected HG-induced exocytosis. Thus, we cannot exclude the possibility that our findings are a MIN6 cell-specific phenomenon. The HG-induced trafficking and fusion event may reflect the observation of “newly recruited granule fusion”, which was previously shown by imaging analysis of insulin exocytosis with TIRF microscopy [11], [19]. Recent studies with TIRF imaging or measurements of membrane capacitance suggested the existence of two phases of insulin granule exocytosis, and that transient first-phase exocytosis with the previously docked granules and sustained second-phase with the newly recruited granules are regulated distinctly by the molecular machinery consisting of different syntaxins and calcium channels [20], [21]. Unfortunately, the TIRF imaging does not correspond directly to electrophysiological analysis as well as biochemical insulin secretion assays, and has also some limitations in quantification. Our method using the multi-labeling reporter system links biochemical assays with imaging analysis, which allows integration of insulin protein age and cell surface exocytotic processes.

## Supporting Information

Figure S1 **Insulin-HT distribution in another stable cell clone.** A, Two independent MIN6/insulin-HT stable cells (clone #19 and #24) were incubated with 5 µM HT-TMR probe for 30 minutes. The cells were then fixed and stained with anti-secretogranin III and Alexa488-conjugated anti-rabbit IgG antibodies. Fluorescent images were captured by confocal microscopy. Bar, 10 µm. B, MIN6/insulin-HT cells (clone #19) were incubated with HT-TMR probe following HT-R110 as described in Fig. 3A. Fluorescent signals of R110 and TMR were observed by confocal microscopy in sequential z-axis planes. The center and bottom image are depicted.
(PDF)

Figure S2 **Monitoring of biosynthetic pathway of insulin-HT in live cells.** MIN6/insulin-HT stable cells (clone #67) were treated with blocking probe for 1 hour. After removal of excess probe, the cells were labeled with HT-TMR for 1 hour at 15°C. Cells were subsequently chased at 37°C for 15 and 30 minutes with nuclear-staining dye Hoechst 33342.
(PDF)

Figure S3 **Localization of newly or previously synthesized insulin-HT in the cell.** Intracellular localization of new or old insulin-HT was analyzed by confocal microscopy, and full sequential Z-axis pictures are shown (see Fig. 3A).
(PDF)

Figure S4 **Full images of insulin-HT secretion analysis.** High glucose-stimulated secretion of new or old insulin-HT was analyzed by a combination of immunoprecipitation and immunoblotting, and full image films are shown (see Fig. 4B). Immunoblotting with anti-biotin cross-reacts to IgGs and shows several non-specific bands.
(PDF)

Figure S5 **Distribution of insulin-HT in INS-1E cells.** A, INS-1E cells were co-transfected with plasmids encoding insulin-HaloTag (insulin-HT) and insulin-EGFP. After 24 hours, the cells were incubated with 5 µM HT-TMR probe for 30 minutes. Fluorescent images were captured by confocal microscopy. B, INS-1E cells transiently transfected with insulin-HT were treated with blocking probe for 1 hour. After removal of excess probe, the cells were labeled with HT-TMR for 1 hour at 15°C (0 min). The cells were subsequently chased at 37°C for 15 and 30 minutes. Fluorescent images were analyzed by confocal microscopy. C, INS-1E cells transiently transfected with insulin-HT were labeled by HT-TMR probe following HT-R110 probe, as described in Fig. 3A. The fluorescent signals were observed by confocal microscopy in sequential z-axis planes. Bar, 5 µm.
(PDF)

Figure S6 **Distribution of insulin-HT in primary mouse islet cells.** Dispersed mouse islet cells were infected with adenoviruses integrating insulin-HT at MOI of 5 pfu/cell. After infection, they were labeled by HT-TMR probe following HT-R110 probe, as described in Fig. 3A. The fluorescent signals were observed by confocal microscopy in sequential z-axis planes. The periphery and the center images of three islet cells were shown. Bar, 5 µm. B, The periphery rates of new or old insulin-HT was analyzed as in Fig. 3B. Data are shown as the mean ± SEM of two independent experiments.
(PDF)
