# Supplementary figures and images for: Preferential Release of Newly Synthesized Insulin Assessed by a Multi-Label Reporter System Using Pancreatic β-Cell Line MIN6
Source: PLoS One. 2012 Oct 25;7(10):e47921. doi: 10.1371/journal.pone.0047921 (PMC3485036; doi:10.1371/journal.pone.0047921)

Figure S1 Hou et al.

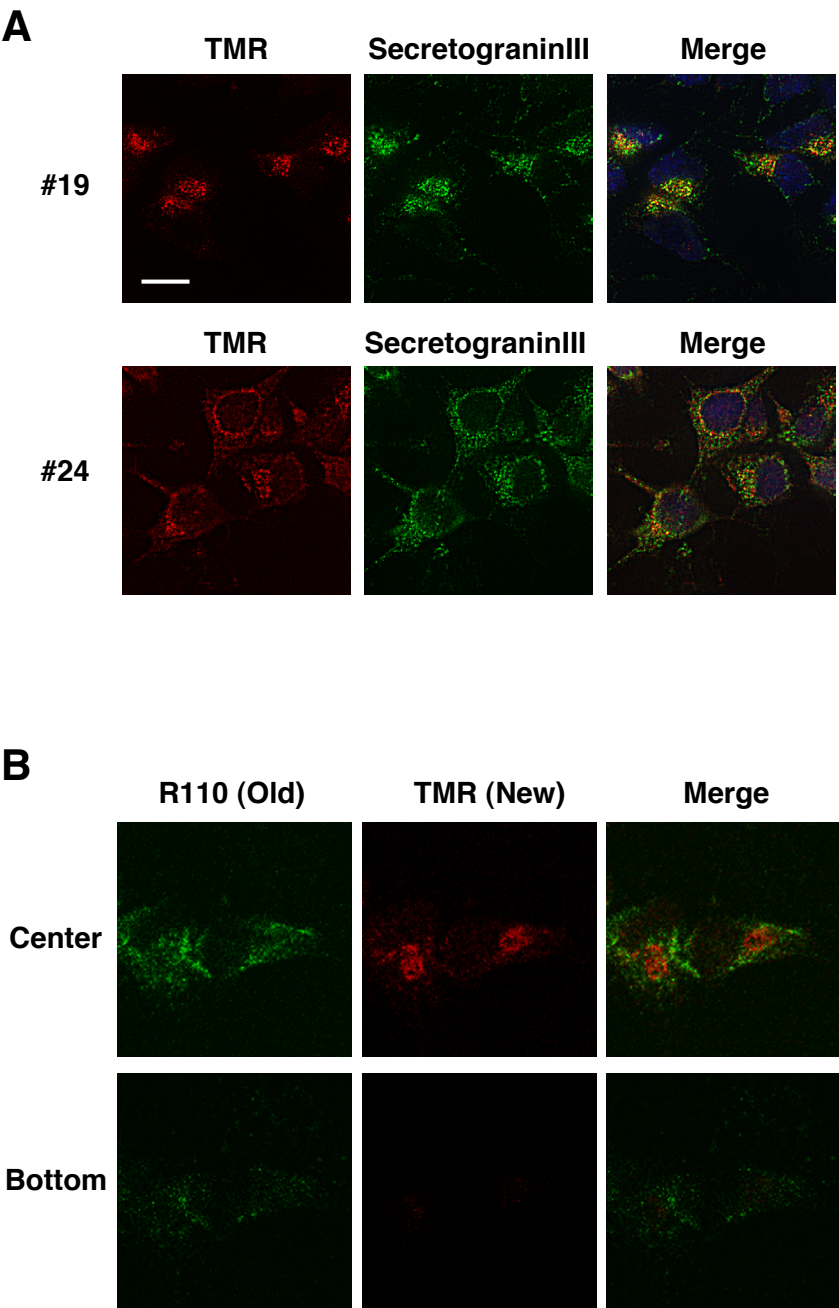

Supplement: Figure S1 — Insulin-HT distribution in another stable cell clone. A, Two independent MIN6/insulin-HT stable cells (clone #19 and #24) were incubated with 5 µM HT-TMR probe for 30 minutes. The cells were then fixed and stained with anti-secretogranin III and Alexa488-conjugated anti-rabbit IgG antibodies. Fluorescent images were captured by confocal microscopy. Bar, 10 µm. B, MIN6/insulin-HT cells (clone #19) were incubated with HT-TMR probe following HT-R110 as described in Fig. 3A. Fluorescent signals of R110 and TMR were observed by confocal microscopy in sequential z-axis planes. The center and bottom image are depicted. (PDF) [file pone.0047921.s001.pdf]

**Figure S2 Hou et al.**

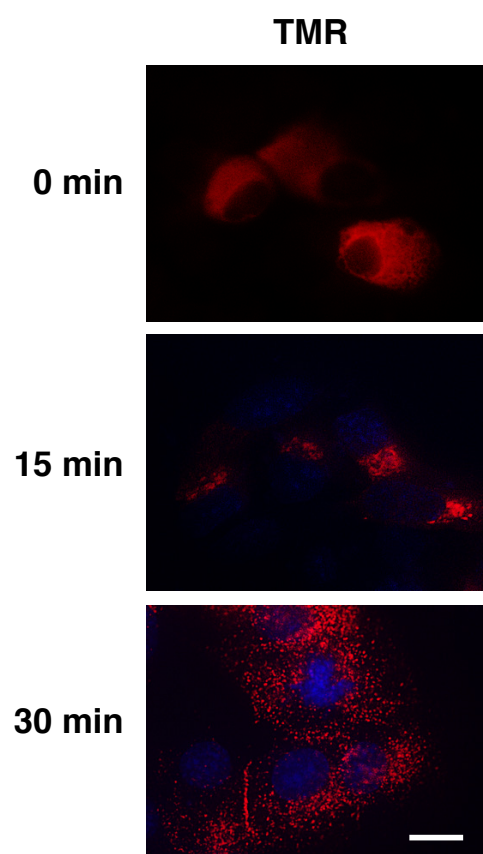

Supplement: Figure S2 — Monitoring of biosynthetic pathway of insulin-HT in live cells. MIN6/insulin-HT stable cells (clone #67) were treated with blocking probe for 1 hour. After removal of excess probe, the cells were labeled with HT-TMR for 1 hour at 15°C. Cells were subsequently chased at 37°C for 15 and 30 minutes with nuclear-staining dye Hoechst 33342. (PDF) [file pone.0047921.s002.pdf]

Figure S3 Hou et al.

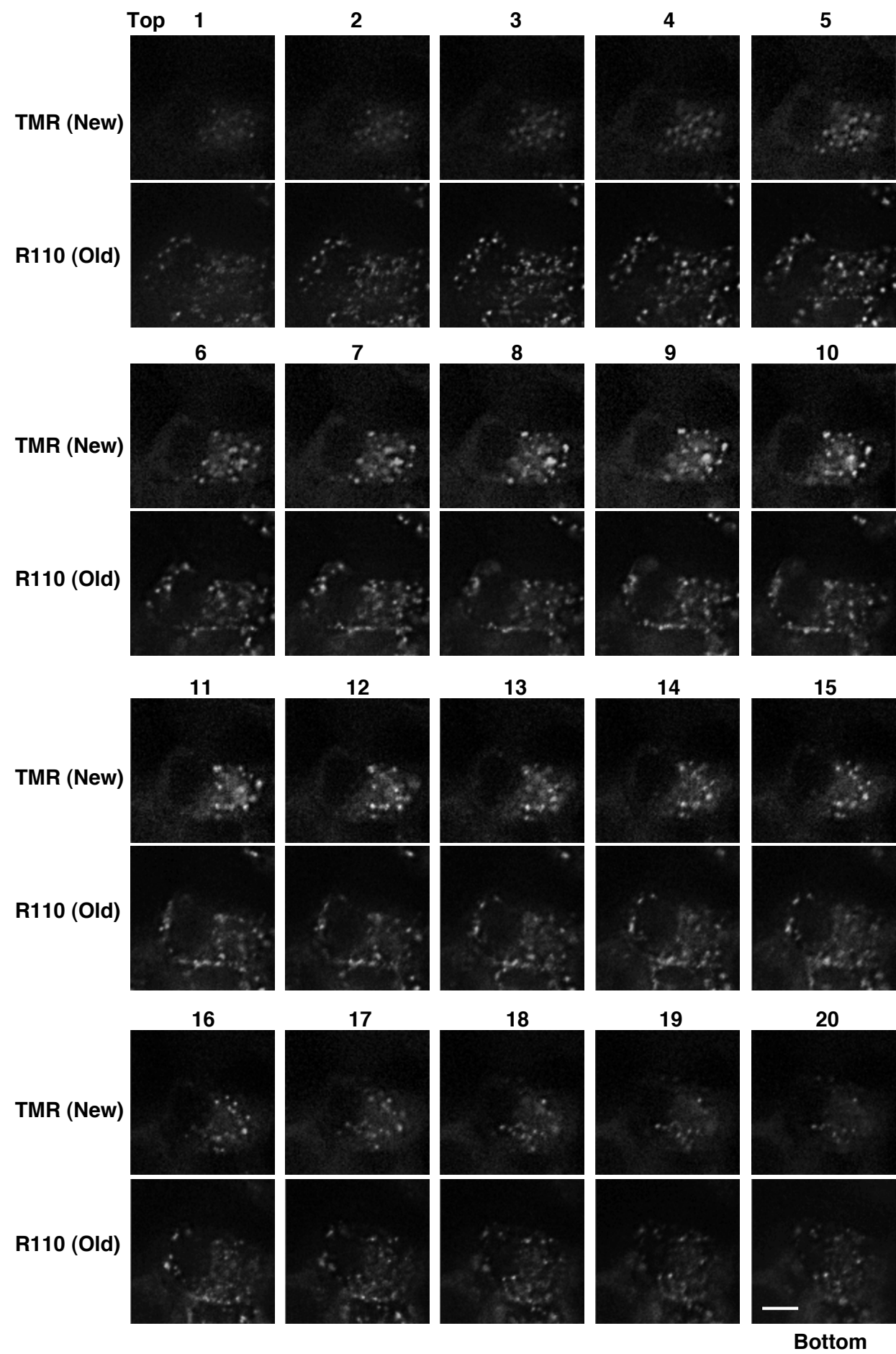

Supplement: Figure S3 — Localization of newly or previously synthesized insulin-HT in the cell. Intracellular localization of new or old insulin-HT was analyzed by confocal microscopy, and full sequential Z-axis pictures are shown (see Fig. 3A). (PDF) [file pone.0047921.s003.pdf]

Figure S4 Hou et al.

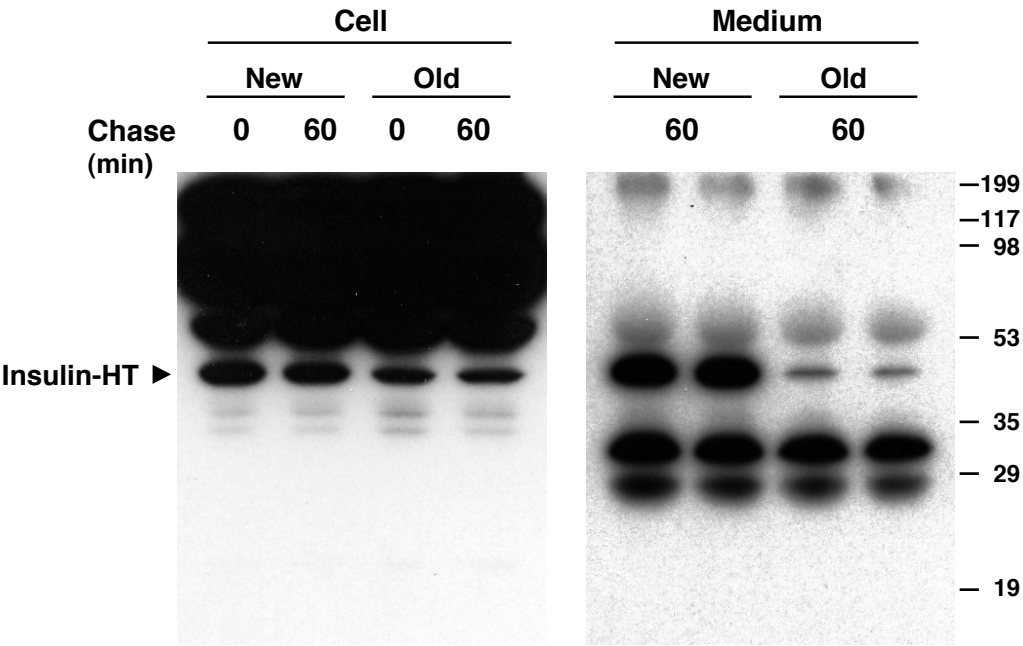

Supplement: Figure S4 — Full images of insulin-HT secretion analysis. High glucose-stimulated secretion of new or old insulin-HT was analyzed by a combination of immunoprecipitation and immunoblotting, and full image films are shown (see Fig. 4B). Immunoblotting with anti-biotin cross-reacts to IgGs and shows several non-specific bands. (PDF) [file pone.0047921.s004.pdf]

Figure S5 Hou et al.

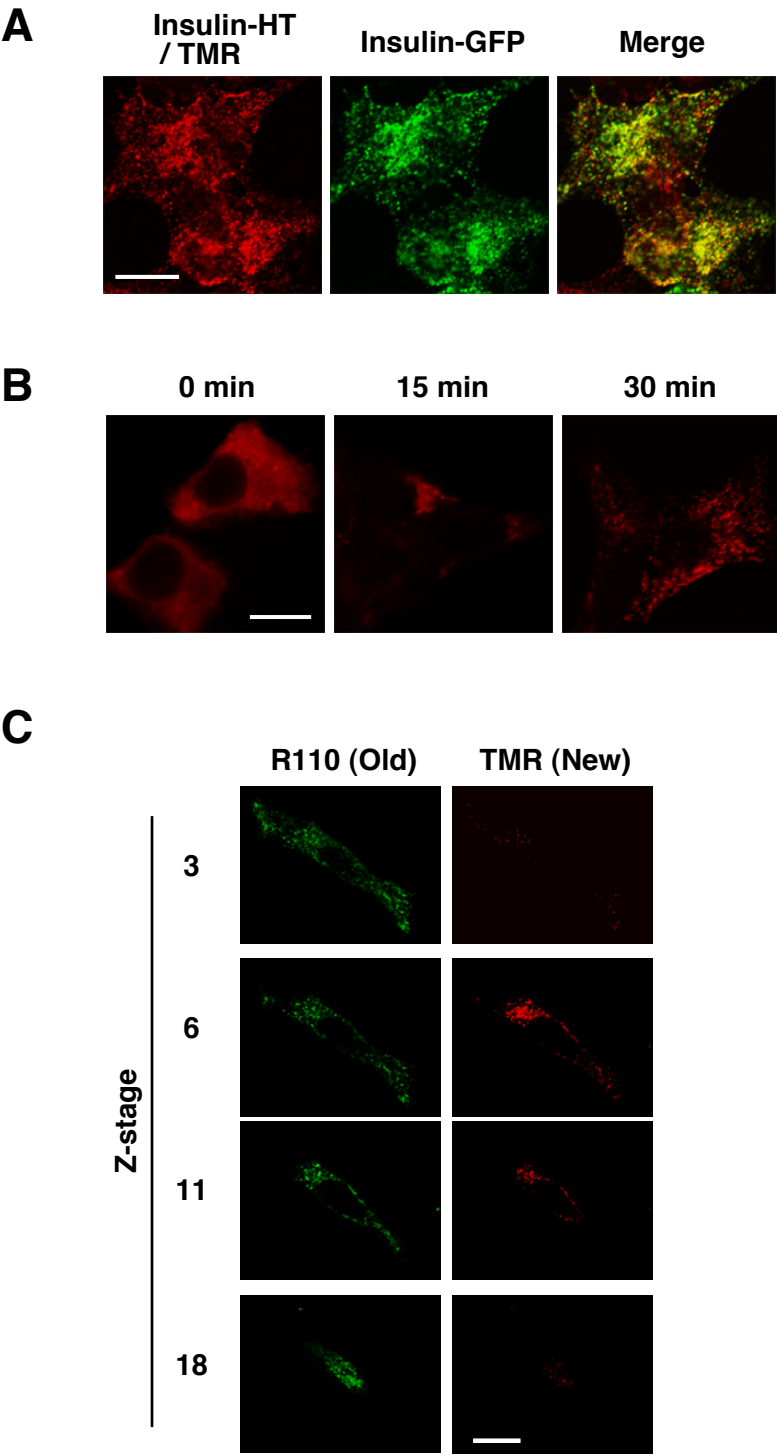

Supplement: Figure S5 — Distribution of insulin-HT in INS-1E cells. A, INS-1E cells were co-transfected with plasmids encoding insulin-HaloTag (insulin-HT) and insulin-EGFP. After 24 hours, the cells were incubated with 5 µM HT-TMR probe for 30 minutes. Fluorescent images were captured by confocal microscopy. B, INS-1E cells transiently transfected with insulin-HT were treated with blocking probe for 1 hour. After removal of excess probe, the cells were labeled with HT-TMR for 1 hour at 15°C (0 min). The cells were subsequently chased at 37°C for 15 and 30 minutes. Fluorescent images were analyzed by confocal microscopy. C, INS-1E cells transiently transfected with insulin-HT were labeled by HT-TMR probe following HT-R110 probe, as described in Fig. 3A. The fluorescent signals were observed by confocal microscopy in sequential z-axis planes. Bar, 5 µm. (PDF) [file pone.0047921.s005.pdf]

**Figure S6 Hou et al.**

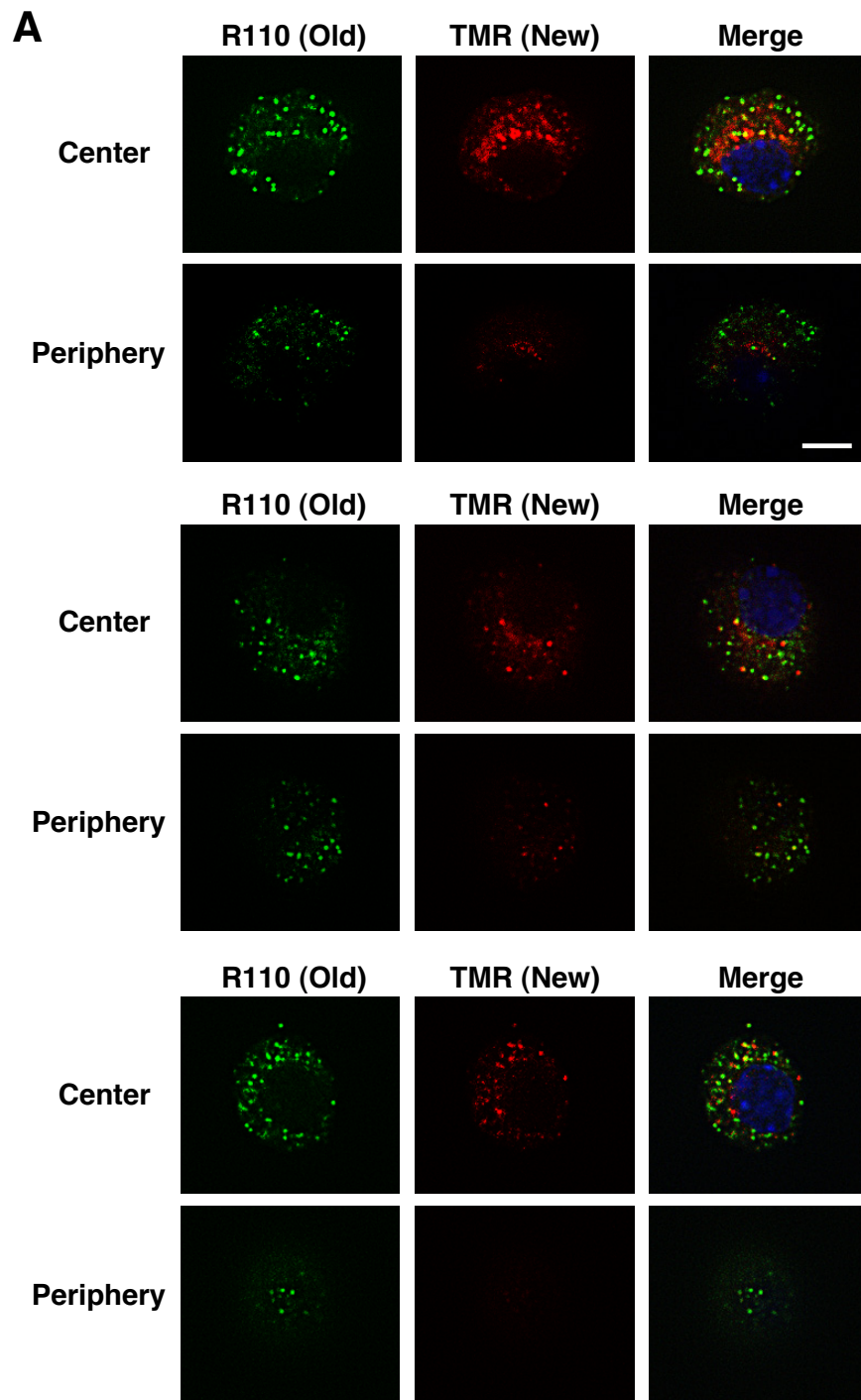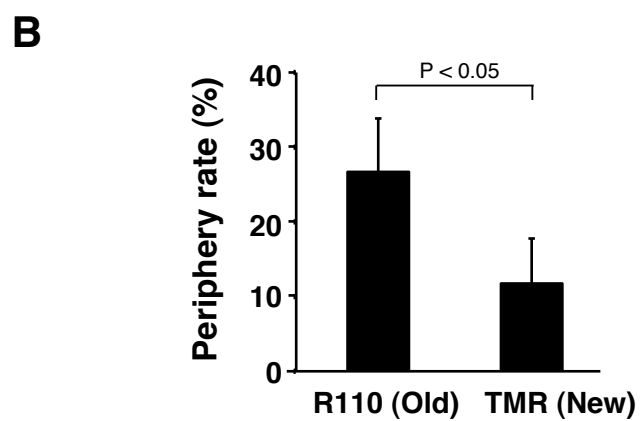

Supplement: Figure S6 — Distribution of insulin-HT in primary mouse islet cells. Dispersed mouse islet cells were infected with adenoviruses integrating insulin-HT at MOI of 5 pfu/cell. After infection, they were labeled by HT-TMR probe following HT-R110 probe, as described in Fig. 3A. The fluorescent signals were observed by confocal microscopy in sequential z-axis planes. The periphery and the center images of three islet cells were shown. Bar, 5 µm. B, The periphery rates of new or old insulin-HT was analyzed as in Fig. 3B. Data are shown as the mean ± SEM of two independent experiments. (PDF) [file pone.0047921.s006.pdf]
